# Popliteal tendon impingement as a cause of pain following total knee arthroplasty: a systematic review

**DOI:** 10.1186/s42836-023-00201-7

**Published:** 2023-09-04

**Authors:** Michael A. Finsterwald, Victor Lu, Octavian Andronic, Gareth H. Prosser, Piers J. Yates, Christopher W. Jones

**Affiliations:** 1 Department of Orthopaedics, Fiona Stanley Hospital, Perth, 6150 Australia; 2 The Orthopaedic Research Foundation of Western Australia (ORFWA), Perth, 6150 Australia; 3 School of Clinical Medicine, University of Cambridge, Cambridge, CB2 0SP UK; 4 Balgrist University Hospital, University of Zurich, 8008 Zurich, Switzerland; 5 Curtin University, Bentley, Perth, 6120 Australia

**Keywords:** Total knee arthroplasty, TKA, Popliteal tendon, Impingement, Popliteus dysfunction

## Abstract

**Introduction:**

Popliteal tendon impingement (PTI) is an under-recognized cause of persistent pain following total knee arthroplasty (TKA). The purpose of the systematic review was to summarize and outline successful strategies in the diagnosis and management of PTI.

**Methods:**

A systematic review following the PRISMA guidelines was performed for four databases: MEDLINE (Pubmed), Ovid EMBASE, Web of Science, and Cochrane Database. It was registered in the International Prospective Register for Systematic Reviews and Meta-analysis (PROSPERO) under the registration number: CRD42023398723. The risk of bias assessment was performed using the criteria of the methodological index for non-randomized studies (MINORS).

**Results:**

A total of 8 studies were included. There were 2 retrospective case series and 6 case reports. The follow-up ranged from 6 to 30 months. Two studies described PTI as an intraoperative phenomenon during TKA with “snapping”; whilst 6 studies described indications and outcomes for arthroscopic tenotomy for PTI following TKA. In making the diagnosis, there was concurrence that the posterolateral pain should be focal and that dynamic ultrasonography and diagnostic injection play an important role. Two specific clinical tests have been described. There was no consistency regarding the need for imaging. There were no reports of instability following popliteal tendon tenotomy or other complications.

**Conclusion:**

PTI should be suspected as a cause for persistent focal pain at the posterolateral knee following TKA. The diagnosis can be suspected on imaging and should be confirmed with dynamic ultrasonography and an ultrasound-guided diagnostic injection. An arthroscopic complete tenotomy of the tendon can reliably alleviate pain and relies on correct diagnosis. There is no evidence for clinically relevant negative biomechanical consequences following tenotomy.

**Level of evidence:**

Systematic Review of Level IV and V studies.

## Introduction

Over 100,000 total knee arthroplasties (TKA) are performed annually for osteoarthritis (OA) in the UK alone [1]. Despite a good outcome for many patients, up to 20% of patients experience chronic pain or dissatisfaction after total knee arthroplasty (TKA). Chronic pain after TKA can affect all dimensions of health-related quality of life and is associated with functional limitations, pain-related distress, depression, poorer general health and social isolation [2].

After significant advancements in implant design were achieved over several decades [3–6], soft-tissue balance and alignment philosophies are the current areas of major clinical research that aim to reduce the number of dissatisfied patients following TKA. Proposed as being more physiological and restoring the three kinematic axes of the knee, there has been a recent increase in the implementation of kinematic alignment as opposed to mechanical alignment [1]. Recognizing the importance of soft-tissue changes is also crucial when dealing with soft-tissue impingement around the knee joint [7].

Popliteal tendon impingement (PTI) is one example of soft-tissue impingement following TKA and may be an under-recognized source of residual pain and poorer outcomes after TKA [8]. Although other causes can be attributed to soft-tissue impingements [9–11], the popliteus tendon is of special interest due to its intraarticular location and its close contact with the posterolateral tibial plateau and/or tibial component and the lateral condylar margin [12]. It originates from the lateral femoral condyle, just distal to the lateral epicondyle, near the origin of the lateral collateral ligament and then inserts in a triangular-shaped fashion along the posteromedial tibial surface [12]. In extension, the popliteus tendon is posterior to the lateral collateral ligament, and in flexion, it resides just anterior to the lateral collateral ligament. The popliteus muscle–tendon unit is a valuable component of the posterolateral corner of the native knee, providing dorsolateral stability. It unlocks the knee and rotates the tibia internally on the femur, as it prevents excessive external rotation of the tibia during knee flexion [13, 14]. The popliteus tendon is characterized by a very high morphological variability [12, 15]: single tendon insertions with variability of the level of insertion or multiple insertions with accessory bands to surrounding structures [16]. It varies in terms of insertion and excursion and can therefore have different anatomical relationships to TKA implants [17].

However, the precise function of the popliteus tendon in patients with TKA is less well-defined. In TKAs, the popliteus may not have the same critical function, as the arthroplasty itself may have more inherent stability in combination with arthroplasty patients often being lower demand. PTI cases have been reported secondary to friction against femoral osteophytes [18] or overhanging prosthetic condyles [19, 20] and have been successfully treated by surgical release. More recent evidence suggests that tibial component positioning and sizing can alter the popliteal excursion even in a well-sized tibial component [21].

In the context of multiple reports describing PTI as an entity responsible for residual pain following TKA, it was the purpose of the current systematic review to summarize and critically appraise the evidence regarding diagnosis as well as surgical management and their outcomes for PTI.

## Methods

### The strategy of the systematic search

The systematic review followed The Preferred Reporting Items for Systematic Reviews and Meta-analysis (PRISMA) guidelines [22]. It was registered in the International Prospective Register for Systematic Reviews and Meta-analysis (PROSPERO) under the registration number: CRD42023398723. A systematic computer-based database search was conducted using CENTRAL (Cochrane Central Register of Controlled Trials), MEDLINE, EMBASE, Scopus, AMED and Web of Science Core Collection. Combinations of the following keywords were used: “popliteus”, “popliteal”, “popliteal tendon” and “impingement”, “pain”, “dysfunction”, with each of the terms: “TKA”, “total knee arthroplasty”, “total knee replacement”. All published studies describing patients with suspected or diagnosed popliteal tendon-related complaints that previously underwent total knee arthroplasty without any demographic limitations from inception until February 13th, 2023 were included in the systematic search.

### Selection process and data extraction

Two authors (OA and VL) performed blind and independent study selection by applying the eligibility criteria. In the cases where consensus could not be reached, a third author (MF) was consulted.

The inclusion criteria were: (1) published peer-reviewed original reports of human studies in English, with a publication date between January 1st, 1973, and March 31st, 2023; (2) a minimum reported level of evidence of IV using the Oxford Centre for Evidence-Based Medicine 2011 Levels of Evidence; (3) patient population included both non-operative and surgically-treated patients with a diagnosis or suspicion of popliteal tendon impingement or dysfunction in total knee arthroplasty.

The exclusion criteria were: (1) review/hypothesis/technique articles or oral presentations; (2) non-English articles; (3) patients who had undergone previous surgery; (4) active inflammatory disease; (5) cadaveric or animal studies. Review articles, surgical techniques, oral presentations, experimental or animal studies, as well as studies mixing and overlapping patient populations were excluded.

### Risk of bias assessment

The risk of bias assessment was performed by two authors using the Methodological Index for Non-randomized Studies (MINORS) criteria [23] for each type of study design. Rating discrepancies were resolved through consensus and consultation with the senior author (MF). MINORS criteria assess eight critical aspects of study design for non-comparative clinical studies and an additional four aspects of study design for comparative clinical studies. Each item is scored zero if the information is not reported, one if the information is reported but inadequate, and two if the information is reported and adequate. Therefore, the maximum possible score is 16 for comparative studies and 24 for non-comparative studies. A scoring system was then used per study such as studies that answered yes to a question from the checklist scored 2, not clear scored 1 and no scored 0. Each score was then converted into a percentage.

### Data synthesis and statistical analysis

The statistical analysis was initially planned to be performed using SPSS (IBM SPSS Statistics, Version 24.0; Chicago, Illinois, USA) and R Software. Although a random-effects model of the meta-analysis was intended as per the PROSPERO protocol, along with a heterogeneity analysis using an I^2^ test, the included studies and collected data were too inconsistent in terms of reported outcomes and did not allow for the performance of a meta-analysis.

## Results

### Study identification

**Fig. 1:**
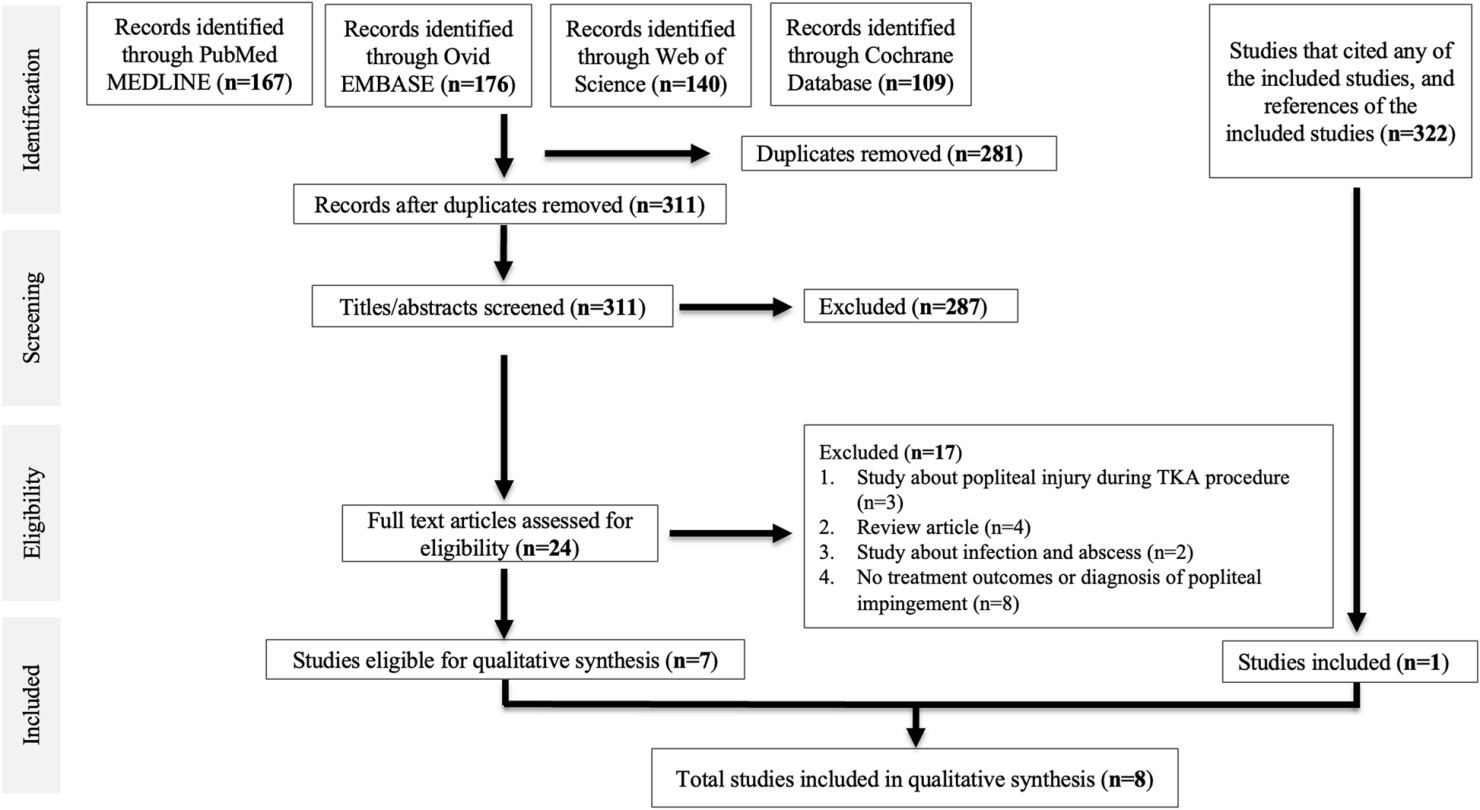
Flowchart of the systematic search

A total of 592 studies could be identified from the initial search of the 4 databases: PubMed/MEDLINE, Ovid/EMBASE, Web of Science and Cochrane Database (Fig. 1). Additionally, 322 reports that cited any of the included studies and the references of the ultimately included studies were also assessed for eligibility.

After the removal of duplicates, 311 studies underwent independent assessment of titles and abstracts assessment by two authors (OA and VL). Full-text assessment of 24 eligible studies resulted in the ultimate inclusion of 8 studies (Fig. 1).

From these, 2 retrospective case series (level IV of evidence) [18, 24] and 6 case reports (level V of evidence) remained [8, 19, 20, 25–27]. There were no prospective studies and no case–control studies that would have used comparators.

### Risk of bias assessment

Due to the limited level of evidence, only 2 retrospective case series [18, 24] enabled the risk of bias assessment using the MINORs tool for non-comparator studies.

Study by Bonnin et al. [24] scored 88%, whilst the case series from Geannette et al. scored 63% [18]. The latter has a higher risk of bias due to the lack of prospective calculation of the sample size, the lack of reporting the loss to follow-up and the lack of prospective data collection (Table 1).

**Table 1:** Individual risk of bias assessment using the MINORS tool (Methodological index for non-randomized studies)

	Adequate statistical analyses	Baseline equivalence of groups	Contemporary groups	An adequate control group	Prospective calculation of the study size	Loss to follow-up less than 5%	Follow-up period appropriate to the aim of the study	Unbiased assessment of the study endpoint	Endpoints appropriate to the aim of the study	Prospective collection of data	Inclusion of consecutive patients	A clearly stated aim	**Total (%)**
Geannette 2018 [18]	NA	NA	NA	NA	0	1	2	1	2	1	2	2	**10/16 (63%)**
Bonnin 2023 [24]	NA	NA	NA	NA	2	1	2	2	2	1	2	2	**14/16 (88%)**

*NA* Not applicable

### Demographics and diagnosis

Overall, the 8 included reports described either diagnostic approaches, treatment outcomes or both, for PTI following TKA for a total of 26 cases (Table 2). The follow-up ranged from 6 to 30 months. The patient’s ages ranged from 47 to 81 years. There were 7 males, 15 females and 4 patients with no reports of their gender.

**Table 2:** Demographics

Author (Year)	Journal	Nr	Follow-up, mean (range or SD)	Age, mean (range)	Gender	MINORS
Allardyce 1997 [19]	*J*. *Arthroplasty*	2	9.5 months (9–10 months)	69	2 M	NA
Barnes 1995 [20]	*J*. *Arthroplasty*	1	n/a	67	1 F	NA
Bonnin 2023 [24]	*KSSTA*	8	18.0 ± 25.8 months	70 (57–81)	8 F	14/16 (88%)
Geannette 2014 [18]	*J*. *Ultrasound Med*.	6	12 months	64 (47–72)	4 M; 2 F	10/16 (63%)
Kazakin 2014 [25]	*J*. *Knee Surg*.	3	24 months	69	1 M; 2 F	NA
Martin 2017 [26]	*Reconstructive Review*	4	6 months	70	1 F; 3 NA	NA
Soejima 2016 [27]	*J*. *Clin*. *Case Rep*.	1	n/a	68	1 F	NA
Westermann 2015 [8]	*Arthrosc*. *Tech*.	1	n/a	n/a	n/a	NA
**Total**	***-***	**26**	**6–30 months**	**47–81**	**7 M; 15 F; 4 unclear**	***-***

*Nr* Number of cases, *NA* Not applicable, *n/a* Not available, *SD* Standard deviation, *M*: Male, *F* Female, *MINORS* Methodological index for non-randomized studies

Two reports [25, 27] have described the preoperative alignment prior to TKA as varus, whilst Bonnin et al. [24] described the indication for TKA (7 medial and 1 lateral knee OA). Geannette [18] has identified one of their PTI cases following a lateral UKR but did not describe the initial alignment or any changes following UKR.

The majority of authors [8, 18, 19] similarly reported the presence of focal posterolateral pain as part of the diagnosis and indication for the management for PTI (Table 3). Kazakin [25] and Barnes [20] described PTI as part of an intraoperative phenomenon that was recognized by the presence of a palpable and/or audible “snapping” of the tendon. Soejima [27] interpreted PTI as the presence of posterolateral pain when flexing the knee beyond 100°. Martin and colleagues described their own clinical test [26]: reproduction of pain when flexing the knee in hip abduction, whilst in a position of lateral decubitus with the affected knee up. Bonnin et al. [24] described their clinical test as reproduction of pain during palpation of the posterolateral area of the joint line at 90° of flexion to full knee extension.

**Table 3:** Diagnostic workup and indications

Author	Implant	Preop Alignment	Avg time from TKA to PTI diagnosis	Etiology	Diagnosis	Imaging	Injection Composition
Allardyce 1997 [19]	n/a	n/a	25 months (3–48 months)	Unclear, observed snapping	(1) Focal pain over the popliteal tendon (2) Snapping is palpable and audible on active ROM	none	n/a
Barnes 1995 [20]	n/a	n/a	intraoperative	Posterolateral impingement against the femoral component	Snapping	none	n/a
Bonnin 2023 [24]	8 HLS-KneeTec, Tornier-Corin PS TKA, France	n/a (but TKA indication was for 7 medial and 1 lateral tibiofemoral OA)	16 months (4–30 months)	Impingement against the superior border of the posterolateral corner of the polyethylene insert	“Popliteus test” The surgeon palpated the posterolateral area of the joint line, whilst the knee was at 90° of flexion, and the patient was asked to extend her knee to full extension. The “popliteus test” was considered positive if the patient experienced excruciating pain as the knee approached full extension	3-tibial baseplate overhang visible on radio-graphs and/or CT scans; 3-PTI on ultrasound	n/a
Geannette 2014 [18]	1 revision fully constrained, 1 lateral UKR, 4 primary PS	n/a	18 months (4–44 months)	osteophyte within the popliteus sulcus of the lateral femoral condyle	(1) Focal pain over the popliteal tendon (2) Positive impingement on dynamic ultrasonography (3) Pain relief after diagnostic/therapeutic injection	6/6-osteophyte PTI on ultrasound 2/6-osteophyte PTI on X-Ray 2/6-osteophyte PTI on MRI	1% lidocaine and 0.25% bupivacaine mixture) and steroid (40 mg of either triamcinolone acetonide or methylpred- nisolone acetate
Kazakin 2014[26]	3 CR	Varus	intraoperative	impingement against the femoral component	Intraoperative snapping during full ROM	none	n/a
Martin 2017 [26]	1/4 Attune PS; 3/4-n/a	n/a	1.6 months	femoral component impingement	PTI test: patient lies in lateral decubitus and holds the affected knee up, flexing the knee in hip abduction from 0 to 90 degrees causes pain	none	n/a
Soejima 2016 [27]	1 Stryker Triathlon PS	Varus	2 months	impingement against the polyethylene insert at 90 degrees of flexion	Pain beyond 100 degrees of knee flexion	none	n/a
Westermann 2015 [8]	1 PS	n/a	n/a	unclear impingement, tendinopathy on arthroscopy	(1) Focal pain with clinical suspicion (2) Failed trial of non-operative management (3) Ultrasound-guided diagnostic injection	none	n/a

*TKA* Total knee arthroplasty, *Preop* Preoperative, *n/a* Not available, *Avg* Average, *OA* Osteoarthritis, *PS* Posterior stabilized, *CR* Cruciate retaining, *PTI* Popliteal tendon impingement, *UKR* Unicompartmental knee replacement, *ROM* Range of motion, *CT* Computer tomography, *MRI* Magnetic resonance imaging

In terms of imaging, only two reports implemented different types of assessment as part of their diagnostic workup (Table 3): conventional X-ray, computer tomography (CT), magnetic resonance imaging (MRI) and dynamic ultrasonography. These were used to assess either the presence of osteophytes or over-sized components [18] or dynamic impingement of the popliteal tendon [24].

Westermann [8] and Geannette [18] were the only authors to emphasize the importance of the amount of pain relief after a diagnostic peri-tendinous injection as a prognostic feature for surgical management. Only 1 study [18] described the composition of the injection that included both a local anesthetic, as well as corticosteroids (Table 3).

### Treatment and outcomes

For all cases that received a diagnosis of PTI postoperatively [8, 18, 19, 24, 26, 27], a knee arthroscopy with debridement and a complete tenotomy, either at the level of impingement or at the level of the femoral insertion was performed (Table 4). There were no reported complications related to the surgical treatment.

**Table 4:** Treatment and outcomes

Author	Sample Size	Follow-up mean (range)	Treatment	Complications	Outcomes
Allardyce 1997 [19]	2	9.5 months (9–10 months)	Arthroscopic debridement and popliteal tendon tenotomy	none reported	complete pain resolution
Barnes 1995 [20]	1	n/a	Tenotomy at the level of femoral insertion (open, intraoperative)	none	n/a
Bonnin 2023 [24]	8	18.0 ± 25.8 months	Arthroscopic debridement and popliteal tendon tenotomy	none	-6/8: complete pain resolution (VAS 0/10); 1/8: partial relief VAS 2/10; 1/8 failed and went to revision TKA for pain and stiffness -OKS range 19–43 -Satisfaction was rated ≥ 80% in 5/7 excluding the revision TKA
Geannette 2014 [18]	6	12 months	Diagnostic/therapeutic injection followed by arthroscopic tenotomy for refractory cases	none reported	-complete pain resolution after injection: 2/6 -complete pain resolution after arthroscopic release: 1/6 -revision TKA: 2/6 -no relief after injection that went on to arthroscopic release and then revision TKA (fully constrained)
Kazakin 2014 [25]	3	24 months	Popliteal tendon tenotomy	none	n/a
Martin 2017 [26]	4	6 months	Arthroscopic debridement and popliteal tendon tenotomy	none	complete pain resolution
Soejima 2016 [27]	1	n/a	Arthroscopic debridement and popliteal tendon tenotomy	none reported	n/a
Westermann 2015 [8]	1	n/a	Arthroscopic debridement and popliteal tendon tenotomy	n/a	complete pain resolution

*n/a* Not available, *VAS* Visual analogue scale, *OKS* Oxford knee score, *TKA* Total knee arthroplasty

Only 4/8 (50%) of authors reported any type of outcome (Table 4). Three case series [8, 19, 26] reported complete pain relief following tenotomy without an objective measurement of outcome and did not use any patient-reported outcomes. Geannette [18] achieved complete pain resolution after injection in 33% (2/6) of cases. Only one had a complete pain resolution after arthroscopic release following a lateral UKR. In two of their cases without any response to injection, the patients went on with revision TKA. The last patient that had marked pain relief following injection underwent an arthroscopic tenotomy but ultimately had a revision TKA to a fully constrained implant. The authors did not report the reason for a constrained revision TKA or whether any instability occurred after the arthroscopic release.

Only Bonnin [24] assessed the outcomes using the Oxford knee score (OKS), the visual analog scale (VAS) and a subjective satisfaction rating. They achieved a satisfaction rate of over 80% in 5/7 patients. The authors had recognized a failed case in their series whereas the patient did not have a positive clinical test preoperatively and complained of global instead of focal pain. The indication was rather based on the presence of tibial baseplate posterior overhang and impingement on dynamic ultrasonography. This case ultimately progressed to revision TKA for pain and stiffness (Table 4).

## Discussion

Although there is a lack of high-level evidence for the management of PTI following TKA for residual pain, our systematic review is the first to have analyzed the existing evidence on both the diagnosis and management modalities for PTI following TKA. Overall, we evaluated a total of 26 cases from 8 different authors utilizing different diagnostic and treatment strategies.

PTI has a multifactorial etiology and the component size, component positioning as well as the presence of mechanical conflict with osteophytes should be evaluated in the first instance. As we observed in our systematic review, PTI can occur in different settings: overhanging or over-sized femoral [20, 25] or tibial components [27], impingement without overhanging or over-sized components[8, 19], impinging against an osteophyte or cementoma [18]. As such, using better-sized implants can potentially avoid impingement and might explain previous clinical investigations that reported better pain scores in patients with “undersized” implants [28], and poorer outcomes in patients with posterior tibial overhang [29]. PTI seems to be able to occur with both varus or valgus preoperative alignment, as was shown in a case of lateral unicompartmental knee replacement (UKR) [18].

**Fig.2:**
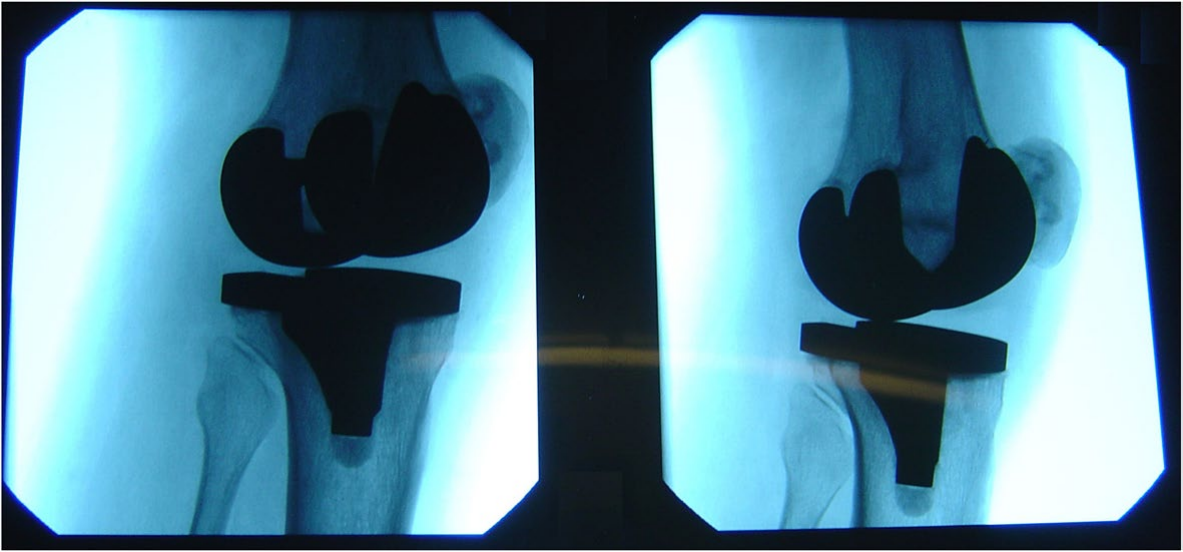
Tibial component overhang confirmed on dynamic fluoroscopy in a patient with popliteal tendon impingement

All authors mentioned the importance of the clinical presentation with focal (not global) posterolateral pain. The validity, sensitivity and specificity of the 2 clinical tests described by Bonnin [24] and Martin [26] are unknown and the utility of these tests is yet to be established. There was no consistency when looking at the need for imaging for assessing PTI. Whilst seeing a potential cause for impingement can suggest the diagnosis on conventional radiographs, CT scans or MRIs, dynamic ultrasonography showing the mechanical conflict in combination with an ultrasound-guided diagnostic injection in the tendon, seems to ensure reproducible results following surgery [8, 18]. Moreover, Geannette et al. reported 2 patients that had complete resolution of symptoms after an image-guided injection and did not require further escalation of treatment [18]. We have also used dynamic fluoroscopy to confirm an overhang of the tibial component for a patient with popliteal tendon impingement (Fig. 2).

After an unsuccessful trial of non-operative management, arthroscopic debridement and tenotomy should be the initial surgical management for suitable candidates. Concern with tenotomy is that it may cause instability in flexion. Cottino et al. [30] reported an increased TKA laxity after popliteal tendon release, both with cruciate-retaining and posterior-stabilized prostheses. However, no instabilities were reported in the studies included in this review. There is an ongoing debate about the clinical importance of the popliteal tendon to stability and patient satisfaction. As opposed to the findings of De Simone et al. [31], who reported lower function scores after popliteal tendon injury in TKA, three other studies [32–34] did not observe any adverse events, either in vivo or in vitro.

Two case reports [20, 25] from our systematic review described intraoperative “snapping” of the tendon during the range of motion as a clinical sign of PTI. The release of the tendon did not affect outcomes at the short-term follow-up, demonstrating that this is an acceptable surgical step when PTI can be observed intraoperatively and no component mal-positioning or incorrect sizing is recognized.

We conclude that the common findings of the authors used for a successful diagnosis of PTI were the following: the presence of focal pain as opposed to global pain, high clinical suspicion of PTI and positive response to diagnostic injection.

### Limitations

We encountered some difficulties in the evidence synthesis due to inconsistency and poor detail when reporting outcomes. The majority of case series reported the outcome as a complete or partial pain relief with only one author using well-defined PROMs such as OKS and VAS [24]. The follow-up in many studies was also heterogenous, but we considered that a minimum of 6 months should have been sufficient in recognizing whether the indication for popliteal tendon release was appropriate and whether instability occurred. Several patients lost to follow-up after a diagnostic/therapeutic injection, further complicating the assessment of its utility [18]. Therefore, an objective assessment of the accuracy of a specific diagnostic test or imaging modality is not possible using the available evidence. Further outcome studies are warranted.

## Conclusion

PTI should be suspected as a cause for persistent focal pain at the posterolateral knee following TKA. The diagnosis can be suspected on imaging and should be confirmed with dynamic ultrasonography and an ultrasound-guided diagnostic injection. An arthroscopic complete tenotomy of the tendon can reliably alleviate pain and relies on correct diagnosis. There is no evidence for clinically relevant negative biomechanical consequences following tenotomy.

## Data Availability

All study-associated data are stored on local storage in the institutional database and is password-protected. These data can be accessed and reused shall the need arise. Requests should be forwarded to the corresponding author.
